# Impacts of brown carbon from biomass burning on surface UV and ozone photochemistry in the Amazon Basin

**DOI:** 10.1038/srep36940

**Published:** 2016-11-11

**Authors:** Jungbin Mok, Nickolay A. Krotkov, Antti Arola, Omar Torres, Hiren Jethva, Marcos Andrade, Gordon Labow, Thomas F. Eck, Zhanqing Li, Russell R. Dickerson, Georgiy L. Stenchikov, Sergey Osipov, Xinrong Ren

**Affiliations:** 1 Department of Atmospheric and Oceanic Science (AOSC), University of Maryland, College Park, Maryland, USA; 2 Earth System Science Interdisciplinary Center (ESSIC), College Park, Maryland, USA; 3 NASA Goddard Space Flight Center, Greenbelt, Maryland, USA; 4 Finnish Meteorological Institute, Kuopio, Finland; 5 Universities Space Research Association, Columbia, Maryland, USA; 6 Laboratory for Atmospheric Physics, Institute for Physics Research, Universidad Mayor de San Andres, La Paz, Bolivia; 7 Science Systems and Applications, Inc., Lanham, Maryland, USA; 8 State Laboratory of Earth Surface Process and Resource Ecology, College of Global Change and Earth System Science, Beijing Normal University, Beijing, China; 9 Division of Physical Sciences and Engineering, King Abdullah University of Science and Technology, Thuwal, Saudi Arabia; 10 NOAA Air Resources Laboratory, College Park, Maryland, USA

## Abstract

The spectral dependence of light absorption by atmospheric particulate matter has major implications for air quality and climate forcing, but remains uncertain especially in tropical areas with extensive biomass burning. In the September-October 2007 biomass-burning season in Santa Cruz, Bolivia, we studied light absorbing (chromophoric) organic or “brown” carbon (BrC) with surface and space-based remote sensing. We found that BrC has negligible absorption at visible wavelengths, but significant absorption and strong spectral dependence at UV wavelengths. Using the ground-based inversion of column effective imaginary refractive index in the range 305–368 nm, we quantified a strong spectral dependence of absorption by BrC in the UV and diminished ultraviolet B (UV-B) radiation reaching the surface. Reduced UV-B means less erythema, plant damage, and slower photolysis rates. We use a photochemical box model to show that relative to black carbon (BC) alone, the combined optical properties of BrC and BC slow the net rate of production of ozone by up to 18% and lead to reduced concentrations of radicals OH, HO_2_, and RO_2_ by up to 17%, 15%, and 14%, respectively. The optical properties of BrC aerosol change in subtle ways the generally adverse effects of smoke from biomass burning.

Biomass burning emits large amounts of black carbon (BC) and organic carbon (OC) particles into the atmosphere. This has profound effects on Earth’s radiation budget and on atmospheric photochemistry. Most current aerosol models treat all OC from biomass burning as purely scattering, thus underestimating heating effect of the total carbon (OC + BC), the primary absorbing component of carbonaceous aerosols^1^. However, recent studies^2^,^3^,^4^,^5^,^6^ suggest that the light absorbing component of OC known as “brown carbon” (BrC) is capable of enhancing total absorption efficiency of OC, altering direct radiative forcing (DRF) at top of the atmosphere from negative to positive^6^,^7^. Our ‘assumed BrC’ is defined by the retrieved total absorption minus the retrieved absorption by the BC component. Measuring BrC absorption is also essential due to its effect on photolysis rates^8^,^9^ and solar UV radiation reaching the surface^10^,^11^,^12^ with important implications for plant growth^13^,^14^ and human health^15^.

Field measurements of light absorption by aerosols (defined as column effective imaginary part of the complex refractive index, *k*) in the visible and near-infrared (NIR) wavelengths (440, 670, 870, and 1020 nm) are available from ~400 global locations of the Aerosol Robotic Network (AERONET)^16^,^17^. Schuster *et al*.^18^ used those measurements to infer the column-averaged mass density and mass absorption efficiency (MAE) of BC at selected AERONET sites. Arola *et al*.^19^ advanced the method of Schuster *et al*.^18^ to infer both BC and BrC volume fractions from the AERONET inversions that show enhanced values of *k* at 440 nm relative to the red and NIR wavelengths. The spectral dependence of BrC absorption can vary strongly depending on the type of aerosol emissions (fossil fuel combustion versus biomass burning), as well as physical-chemical transformation (primary versus secondary organic aerosols)^6^,^20^. As a result, the 440 nm wavelength does not have enough sensitivity to properly detect some types of BrC. We combine AERONET almucantar inversions in the visible–NIR wavelengths with diffuse and direct surface irradiance measurements from UV Multifilter Rotating Shadowband Radiometer (UV-MFRSR)^11^,^21^ to estimate the BrC column mass density; this is not possible from standalone AERONET inversions. Previous measurements of column aerosol absorption in UV wavelengths have been conducted in urban/suburban locations^21^,^22^,^23^. We present the first measurements of the spectral dependence of *k* for smoke down to the biologically active, UV-B wavelengths (~305 nm) to estimate the MAE of BrC, the BrC/BC column mass density ratio, and effects on tropospheric ozone photochemistry.

To measure UV absorption by smoke, we conducted a field campaign in Santa Cruz, Bolivia in September 2007, the month of peak carbonaceous aerosol production in South America from agricultural biomass burning in the Amazon Basin^24^,^25^, using UV-MFRSR irradiance measurements. The total (scattering and absorption) aerosol optical depth measured by AERONET at 440 nm ranged from 0.74 to 2.27. Satellite measurements of UV aerosol absorption optical depth (AAOD) from Ozone Monitoring Instrument (OMI) on board NASA’s Earth Observing System Aura satellite^26^,^27^ (see Supplementary Section 2 for details) clearly show elevated AAOD values over Santa Cruz in September 2007 (Fig. 1a). The flow of smoke was blocked by the Andes Mountains (see the true color satellite imagery in Fig. 1b), so smoke accumulated over the area to the east of the mountain range. This increased the fraction of aged brown secondary organic aerosol, with less absorption at visible wavelengths but stronger absorption in the UV^5^,^28^. Biomass burning in the tropics also produces elevated levels of tropospheric ozone, a secondary pollutant resulting from photochemical reactions involving nitrogen oxides (NO_x_ = NO + NO_2_) and volatile organic compounds (VOC) in the presence of sunlight^29^,^30^. Tropospheric ozone negatively affects agricultural crops, the human respiratory system, and is the main culprit responsible for photochemical smog.

## Results

### Retrieval and evaluation of spectral dependence of smoke absorption

**Figure 2:**
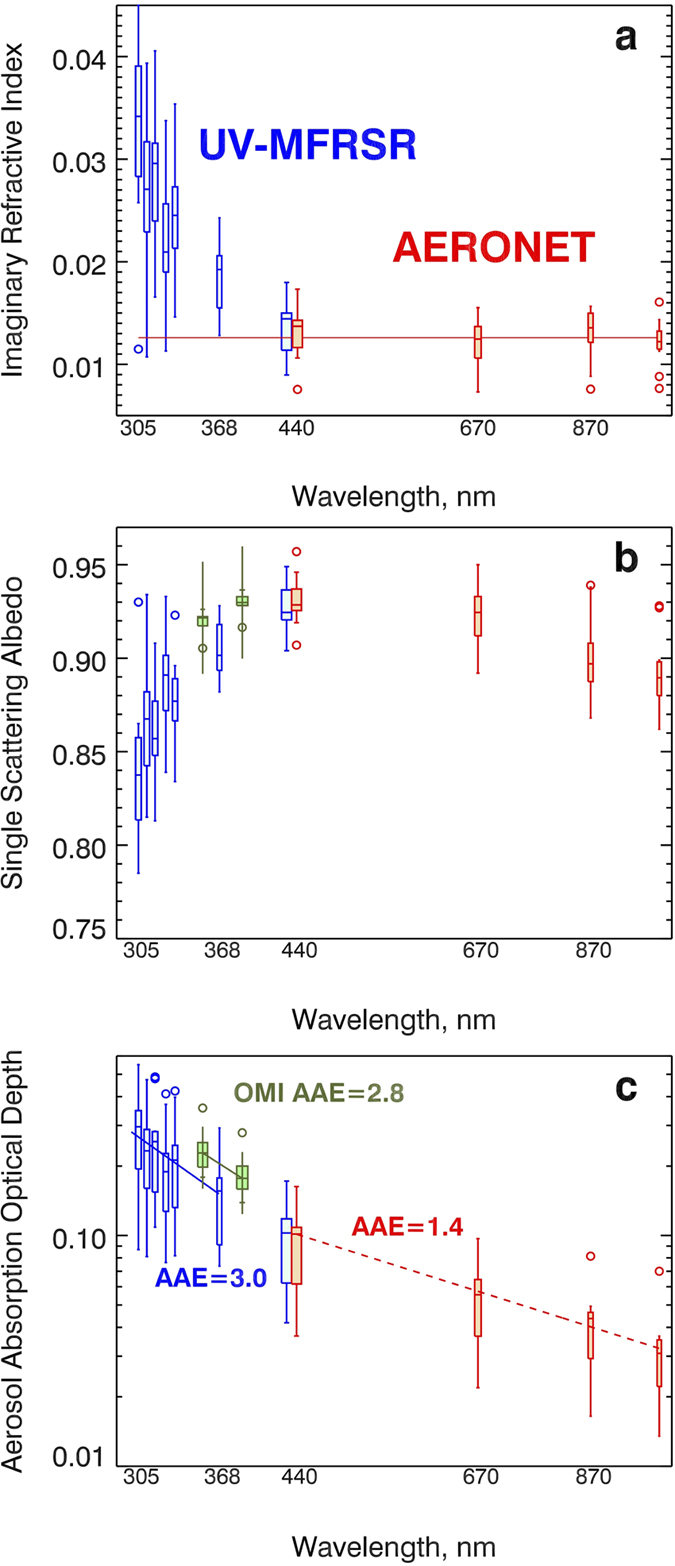
Spectral dependence of smoke aerosol absorption parameters derived from ground-based and satellite (OMI) retrievals during the field campaign in Santa Cruz, Bolivia in September-October 2007. (**a**) Imaginary part of the column effective refractive index (*k*), (**b**) Single scattering albedo (SSA), (**c**) Aerosol absorption optical depth (AAOD = AOD*(1-SSA)). Retrievals are from UV-MFRSR (blue symbols), Vis–NIR AERONET (red symbols), and satellite OMI UV (green symbols). All retrievals are shown as box-whisker plots. Boxes are the interquartile range (IQR; 25 to 75 percentiles) and whiskers are stretched to the maximum and minimum within 1.5 times the IQR. The circles show the outliers. The solid red line in a shows the theoretically calculated campaign–average *k* assuming that BC is the only absorbing component. The error bars in b and c for OMI-retrieved SSA and AAOD (±0.03 for SSA and ±30% for AAOD) are shown as thin vertical lines exceed the whisker’s range.

Figure 2a shows joint UV-MFRSR and AERONET retrievals of spectral *k* (see details in Supplementary Section 1). Our assumptions about mixing state, size distribution, and sphericity are fully consistent with the standard AERONET inversions^17^,^31^. We only use sphericity retrievals by AERONET exceeding 95% to justify the sphericity assumption. Both retrievals agree at 440 nm. The flat spectral dependence of *k* in the visible–NIR region is consistent with a “BC only absorption” assumption^18^,^32^. However, this hypothesis (see the extrapolated red line in Fig. 2a) is grossly inconsistent with the observed enhanced values of *k* in the UV. We postulate that the enhanced *k* values in the UV are a manifestation of the presence of a selectively UV absorbing BrC component of the aged carbonaceous aerosols from tropical forest burning^5^,^33^.

To compare with OMI retrievals at 354 nm and 388 nm, we convert the UV-MFRSR-retrieved *k* into single scattering albedo (SSA) assuming AERONET inferred aerosol size distribution and spherical aerosol shape (AERONET spherical particles fraction > 95%) (Fig. 2b). The average UV-MFRSR SSA at 388 nm (linearly interpolated from retrievals at 368 nm and 440 nm) is slightly smaller than the OMI values, but within the 0.03 error typically assumed in OMI retrievals^27^. Fig. 2c shows derived spectral dependence of AAOD, which describes the total column smoke absorption optical depth due to both BC and BrC. The UV-MFRSR AAOD values agree well with AERONET AAOD at 440 nm and are within the ±30% error bar of the OMI-retrieved AAOD at UV-A wavelengths (Fig. 2c).

Similarly to the Angstrom Exponent (AE), which characterizes spectral dependence of AOD parameterized by power law, the absorption Angstrom Exponent (AAE) characterizes spectral dependence of AAOD = AOD*(1-SSA). For smoke aerosols, there is no single power law parameterization describing AAOD spectral dependence from UV to the NIR wavelengths. This results in spectrally dependent values of AAE. The UV-MFRSR derived AAE in UV wavelengths (~3.0 in the range 305–368 nm) agrees well with the OMI assumed AAE (~2.8 between 354 nm and 388 nm)^34^, which is significantly larger than the AAE in the visible–NIR wavelengths (AAE ~1.1 ± 0.3 for BC only absorption)^35^. This confirms the general assumption that smoke absorption in the visible–NIR wavelengths is dominated by BC component, while in the UV band absorption by BrC plays a significant role. We calculated AAE_BrC_ by fitting a power law equation to the retrieved values of BrC absorbing optical depth (AAOD_BrC_) from 305–368 nm. AAOD_BrC_(λ) = total AAOD(λ) −AAOD_BC_(λ) (See Table 1) where AAOD_BC_ is calculated assuming a constant refractive index from the AERONET retrievals at 440 nm. Our estimated AAE_BrC_ falls within the range of the previous measurements, *e.g.* 5.1 ± 1.9 in Indo-Gangetic Plain^36^, 5.83 ± 0.51 in Beijing^37^, and slightly less than the higher values of 6–7 measured for humic-like substances in the Amazon basin^38^. This confirms the spectral dependence that we believe describes South American forest burning BrC optical properties in chemistry- and aerosol- transport models.

### Estimating the BrC volume fraction and BrC/BC ratio

Arola *et al*.^19^ inferred BrC volume fraction (f_BrC_) in addition to BC volume fraction (f_BC_), but only for cases when the AERONET retrieved *k* value at 440 nm is enhanced, compared to the longer visible and NIR wavelengths (670–1020 nm). This enhancement was attributed to additional BrC absorption and allowed the calculation of f_BrC_ using *a priori* information about the complex refractive index of BrC from previous measurements. The method uses Maxwell-Garnett (MG) mixing rule assuming an internal mixture of BC and BrC embedded in non-absorbing host. The assumed refractive indices for these components are given in Arola *et al*.^19^. Our measurements show no evidence of enhanced BrC absorption at visible wavelengths (*i.e.*, insignificant *k* enhancements at 440 nm compared to longer wavelengths, Fig. 2a), but significantly higher *k* in UV. Therefore, we advance the approach of Arola *et al*.^19^ to estimate f_BrC_ using enhanced *k* values at a longer UV-A wavelength (*i.e.*, 368 nm) which is not available from AERONET inversions. We also reduce the lower limit of the *a priori* BrC imaginary refractive index at 368 nm, *k*_BrC-368_ = 0.025, from recent smoke chamber experiments^6^. The upper limit on *k*_BrC-368_ = 0.14 is assumed from laboratory measurements of more absorbing African smoke samples^2^ (see Supplementary Fig. S1). Consequently, we calculate a range of values of f_BrC_ from ~0.05 (using upper limit of *k*_BrC-368_)^2^ to ~0.27 (using lower limit of *k*_BrC-368_)^6^. We have verified low sensitivity of f_BrC_ to the relative humidity by varying the real part of the refractive index (*n*) of host^39^. For instance, changing *n* by 10% would modify the BrC volume fraction by less than 2%. Assuming BrC mass density^40^ of 1.2 g/cm^3^, we estimate the range of BrC column mass densities from ~11 to 61 mg/m^2^, where the higher limit corresponds to the assumed lower limit of *k*_BrC_^6^ typical for Amazon forest burning smoke (see Methods section for details).

The inferred BrC/BC column mass density ratio ranges from 1.7 to 9.5 (see Supplementary Table S1). Previous studies have reported OC/BC ratios ranging from 4.3 to 12.5 for tropical forests, 8.3 to 16.7 for the Cerrado (South American savanna), and 12.5 to 33.3 for boreal forests^41^. These ratios are larger than our measured BrC/BC ratios because total OC is composed of absorbing (BrC) and non-absorbing OC. Thus, the ratio of BrC to BC is expected to be smaller than the ratio of total OC to BC. The primary sources of aged smoke in Santa Cruz are Cerrado and Amazon basin tropical forest burning, which is done for the purpose of clearing land for agricultural use, and the burning of crop residue after harvesting on existing agricultural land^42^. The highest smoke concentrations measured downwind at Santa Cruz are likely from tropical forest burning^42^. Our maximum estimated BrC/BC ratio (~9.5 using lower limit of *k*_BrC_ values from Saleh *et al*.^6^) is consistent with the previous measurements for tropical forest and Cerrado burning. The lower limit of BrC/BC (~1.7) was obtained by assuming the upper limit of *k*_BrC-368_ from laboratory measurements of African savanna smoke samples collected on filters during the Southern African Regional Science Initiative (SAFARI 2000)^2^. A good agreement is found between BrC/BC and the OC/BC ratio (1.7) measured from agricultural land^41^.

### Enhanced BrC spectral absorption in UV

**Figure 3:**
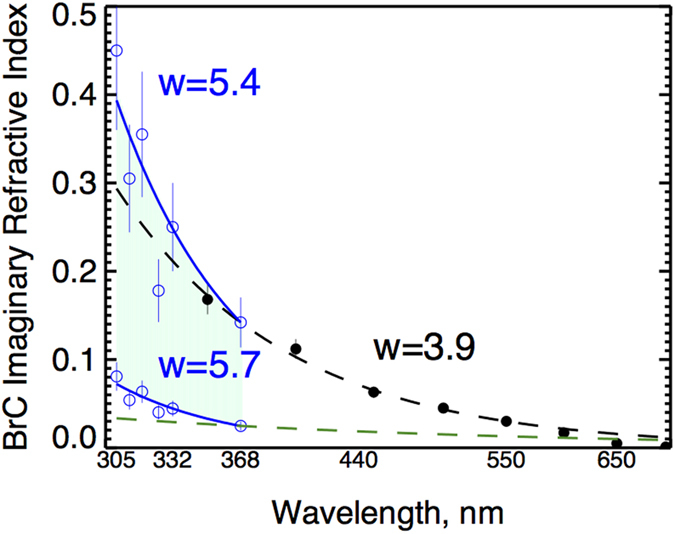
The inferred spectral dependence of BrC imaginary refractive index (*k*_BrC_) in UV (blue circles with 20% error bars). Black circles show the upper limit for *k*_BrC-368_ values derived from African savanna burning samples (10% uncertainty)^2^. Lower limit of *k*_BrC-368_ parameterization (green dashed lines) is based on smoke chamber experiments^6^ showing low spectral dependence (w = 1.6). The shaded area shows the variability range in our inferred *k*_BrC_ in the UV wavelengths. We inferred much larger spectral dependence (w ~ 5.4–5.7) in the UV-B than previously reported (~1.6**–**4) in longer UV and visible wavelengths^2^,^6^.

Previous measurements^2^,^6^ of spectral absorption in the UV do not extend to biologically important wavelengths shorter than 350 nm. We use BrC volume fraction derived at 368 nm to infer spectral dependence of *k*_BrC_ at shorter, more biologically active UV-B wavelengths down to 305 nm (Fig. 3). Specifically, we partitioned the retrieved *k*_ret_ into its *k*_BrC_ and *k*_BC_ components assuming that the smoke layer can be represented by a mixture of BC and BrC embedded into a non-absorbing host. The partitioning is carried out by applying the MG mixing rule to express the measured effective imaginary refractive index (*k*_ret_), in terms of the volume fractions of BC (f_BC_) and BrC (f_BrC_) and their complex refractive indices (*k*_BC_ and *k*_BrC_). The real part of the refractive index for the non-absorbing host, BC, and BrC is assumed to be spectrally constant as in Arola *et al*.^19^ Following this approach, *k*_BrC_ values at 305, 311, 317, 325, and 332 nm are calculated by fitting the retrieved spectral *k*_ret_ using the MG mixing rule with the previously calculated and spectrally independent f_BC_, f_BrC_, and *k*_BC_ (see Methods for details). The calculated spectral dependence of *k*_BrC_ in the UV-B (characterized by the power exponent, w = 5.4–5.7) is significantly stronger than previous laboratory estimates, e.g. by Kirchstetter *et al*.^2^ (w = 3.9) and smoke chamber experiments by Saleh *et al*.^6^ (w = 1.6) (see Fig. 3). Thus, our measurements show that BrC in aged Amazonian smoke absorbs UV solar radiation with stronger spectral dependence than previously reported. This result has important implications for tropospheric photochemistry and biological effects of UV-B as discussed next.

We further convert our calculated AAOD for BrC (AAOD_BrC_) and column mass density for BrC into specific mass absorption efficiency for BrC (MAE_BrC_) in the UV wavelengths (Table 1). MAE_BrC_ is a useful parameter to compare with laboratory measurements and can be directly used in aerosol transport models. It has not been previously measured in the field under smoky conditions. The derived MAE_BrC_ spectral dependence for smoke shows a strong increase at the shorter UV-B wavelengths similarly to those previously measured in anthropogenic aerosols^43^. The maximum and minimum values of MAE_BrC_ (Table 1) are associated with the assumed range of *k*_BrC-368_^2^,^6^. Our minimum values (MAE_BrC_ = 0.9–2.9 m^2^/g) are comparable with the previous measurements of low absorbing organic compounds in smoke from different locations, such as Alaska^44^, Siberia^44^, Indo-Gangetic Plain^45^, Beijing^37^, Colorado^4^, and Amazon basin^38^. Our maximum values (MAE_BrC_ ~5–16 m^2^/g) are consistent with previous measurements for more absorbing organic aerosols originated from urban pollution^43^ and savanna burning^2^. The different ranges of MAE_BrC_ for different wavelengths (See Table 1) suggest that BrC absorption should be treated regionally in chemical transport models. The upper limits of MAE_BrC_ ranges should be used in areas where there is savannah burning. The lower limits of MAE_BrC_ ranges should be used in areas where BrC is expected to be less absorbing. For Santa Cruz, the lower limits are more appropriate.

### BrC absorption effect on surface UV and photochemistry

**Figure 4:**
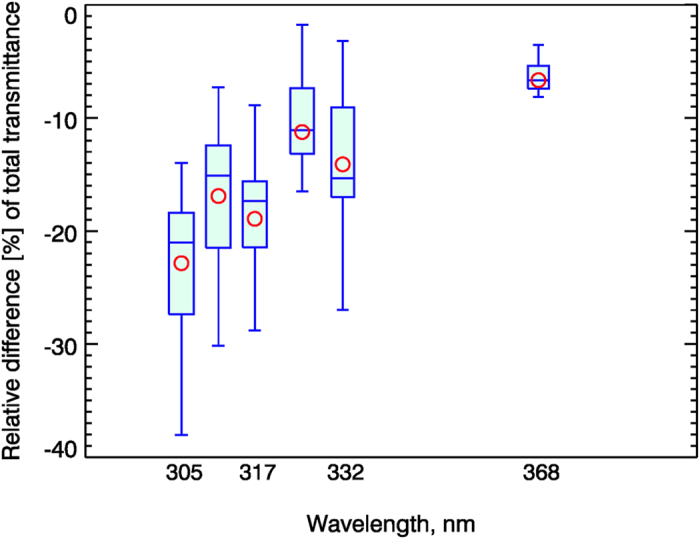
Enhanced BrC absorption causes 20% decrease in the most damaging short wavelength surface UV-B irradiance (305–320 nm) . Box-whisker plots show the relative difference [%] between our measured surface spectral UV (BC plus BrC absorption) and model (assuming BC only) surface UV: (UV_meas_−UV_BC_)/UV_BC_ × 100%. Red circles show independent model estimates using different LibRadtran (http://www.libradtran.org) RTM for the fixed SZA (45°) and ozone column amounts (272 DU).

Excessive exposure to UV-B radiation causes damage to the eyes, suppression of the immune system, photoaging, and skin cancer^15^,^46^. There is also mounting evidence that it alters plant development and growth^13^,^14^. Under unpolluted and clear sky conditions, the levels of surface UV are high in Santa Cruz because of low overhead ozone and low solar zenith angles. Thus, the enhanced absorption of BrC at the most damaging UV-B wavelengths might play a role as a natural sunscreen. To quantify the specific spectral reduction of the surface UV due to absorption by BrC, we performed radiative transfer simulations of the spectral surface UV with and without BrC absorption. Figure 4 shows that the effect of BrC absorption causes an additional 20–25% reduction at the most damaging UV-B wavelengths reaching the surface (*i.e.,* 305 nm) compared to the BC only (spectrally flat) absorption. This previously unaccounted reduction in surface UV-B irradiance is important for health risk assessments^15^,^46^ and estimating UV-B effects on crop yield^13^,^14^. Unaccounted absorption by BrC can also explain the positive bias in satellite derived surface UV-B compared to ground-based measurements^11^,^47^. The BrC absorption effect is smaller at longer UV-A wavelengths (~10% reduction at 320–330 nm).

**Figure 5:**
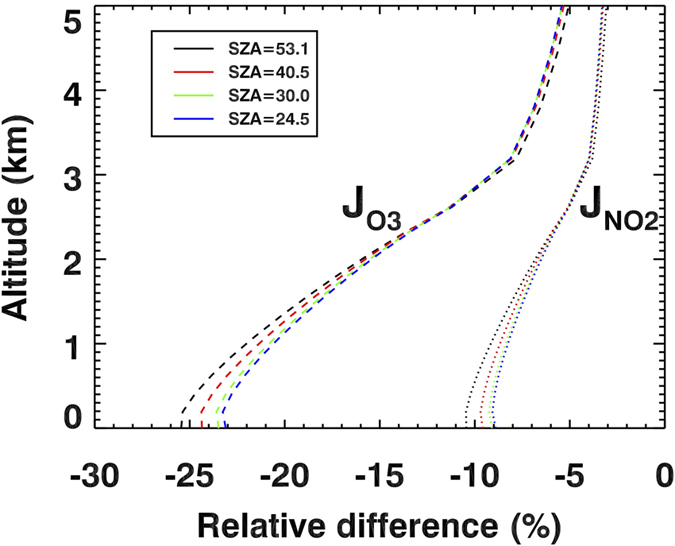
Modeling the impact of BrC absorption on the rate of tropospheric ozone production. The vertical profiles show relative differences [%] in photolysis rates for NO_2_ (J_NO2_: dotted line) and ozone to O(^1^D) (J_O3_: dashed line) due to enhanced BrC UV absorption for different SZAs using a radiative transfer model. We assumed a homogeneously distributed smoke layer below 3 km as measured by space-based lidar. The ozone loss mechanism linked to J_O3_ is more significantly reduced than the production mechanism linked to J_NO2_. Input *k*_ret_ values for calculating photolysis rates are described in Supplementary Table S2.

The stronger absorption of BrC at UV-B wavelengths affects the atmospheric photochemistry. Dickerson *et al*.^8^ and other studies^48^,^49^ show that carbonaceous aerosols reduce the photolysis rate of NO_2_ (J_NO2_) responsible for ozone production by 10–30%. In extreme cases, J_NO2_ is reduced by 70% near the ground, while it is increased by 40% above the smoke layer due to aerosol backscattering^50^. Yet, these studies^48^,^49^,^50^ did not have the information necessary to discriminate between BC and BrC absorption. Our measurements allow us to isolate the effect of BrC absorption on photolysis rates (see Supplementary Fig. S2 for details) and ozone production using a radiative transfer model and a chemical box model (see Supplementary Section 3 and 4 for details). The enhanced absorption of BrC at the UV-B wavelengths around 308 nm (Fig. 3) decreases the photolysis rate of O_3_ (J_O3_) linked to the OH production by ~25% at the surface relative to BC only absorption (Fig. 5). But smaller absorption at longer UV wavelength (390 nm) decreases J_NO2_ linked to the ozone production mechanism by only 10%. Photolysis of carbonyls such as aldehydes is also inhibited by BrC, and this slows production of radicals OH, HO_2_, and RO_2_. Our box chemical model runs considering all major species subject to rapid photolysis (e.g., HCHO, CH_3_CHO, CH_3_OOH, HONO, H_2_O_2_, CHOCHO, CH_3_COCHO, CH_3_COONO_2_) reveal that the optical effects of BrC inhibit net surface and lower tropospheric ozone production by up to 18% (see Supplementary Fig. S3b). A recent study^51^ also reports that the absorption of BrC decreases OH concentration in South America in September by up to 35%. The reduced ozone and VOC photolysis rates due to BrC reduce concentrations of radicals OH, HO_2_, and RO_2_ by up to 17%, 15%, and 14%, respectively (see Supplementary Fig. S4), which in turn, produces less surface ozone. The reduction in the HO_x_ (OH + HO_2_) and RO_2_ concentration also decreases the rate of removal of many other trace gases.

## Discussion

The combination of surface and space-based remote sensing of aerosol optical properties shows that biomass burning of the Cerrado, Amazonian forest, and agricultural lands in South America generates substantial amounts of light absorbing “brown carbon”, BrC. For the first time we have characterized the wavelength dependence of BrC absorption at the shortest and most biologically and chemically active UV-B wavelengths reaching Earth’s surface under aged biomass burning smoke conditions. The estimated optical properties of this BrC differ significantly from the optical models of BC or OC currently assumed in chemical- and aerosol-transport models. While the strong absorption of UV-B radiation seen for BrC can ameliorate some of the adverse health effects of smoke, biomass burning aerosols remain major environmental and health hazards^52^.

Our estimated range of the column volume fraction of BrC is 0.05 to 0.27 and of its column mass density is ~11 to 61 mg/m^2^. The best estimate is likely close to the upper limit because the lower limit represents more absorbing BrC (therefore, lower mass) from high temperature burn conditions in southern Africa savanna. The mass absorption efficiencies of BrC are largest at 305 nm, but vary significantly (e.g., 2.9 to 16.1 m^2^/g) due to variability in BrC column effective imaginary refractive index, *k*_BrC_. Regardless of this uncertainty in absolute values of *k*_BrC_, we were able to constrain its spectral dependence with improved accuracy. The best fit to the spectral dependence of *k*_BrC_ in UV wavelengths is the power-law with retrieved exponent of 5.4 to 5.7.

Our derived total (BC + BrC) absorption Angstrom Exponent (AAE) in UV wavelengths (~3.0 in the range 305–368 nm) agrees well with the OMI assumed AAE (~2.8 between 354 nm and 388 nm)^34^, validating this *a-priori* assumption regarding *k* spectral dependence for smoke aerosols.

The primary difference between BrC and BC is the strong spectral dependence of the absorption of UV radiation by BrC. In comparison to BC alone, the combination of BC and BrC strongly decreases surface UV-B actinic flux, photolysis rates, and the rates of production of radicals RO_x_ (RO_x_ = OH+HO_2_+RO_2_+RO) and O_3_. Although a complex and nonlinear relationship exists with respect to concentrations of nitrogen oxide precursors, the observed optical properties of BrC reduce the rate of ozone production by up to ~18% over the full range of possible NO_x_ values, from 100 ppb near the fires to 5 ppb well downwind of the fires. Radicals are also produced more slowly, and the lower concentrations of RO_x_ mean longer lifetimes for ozone and its precursors (NO_x_ and VOCs). This could lead to greater ozone concentrations downwind. The net impact will depend on the rate of dispersion and non-linear photochemistry including the speciation of VOCs. Future studies should include sufficient ambient measurements to initialize and constrain a 3-D chemical transport model.

## Methods

Our field measurements of BrC and BC aerosol absorption are based on retrievals of total column effective imaginary refractive index (*k*_ret_) from AERONET inversions at visible and NIR wavelengths^17^ and Diffuse/Direct irradiance inversions at UV wavelengths^11^. First, we derive the BC volume fraction (f_BC_) from AERONET *k*_ret_ at visible and NIR wavelengths from 670 to 1020 nm^18^. Next, we calculate the BrC volume fraction (f_BrC_) assuming (1) flat spectral dependence of *k*_BC_, (2) known value of *k*_BrC_ at 368 nm from laboratory absorption measurements or smoke chamber experiments (see Supplementary Fig. S1). Finally, we use the inferred f_BC_ and f_BrC_ to calculate *k*_BrC_ at short UV-B wavelengths by fitting *k*_ret_ at 305, 311, 317, 325, and 332 nm using MG mixing rule. The details are given below.

### BrC volume fraction calculation

Here we demonstrate our strategy of BrC retrievals combining AERONET and UV-MFRSR inversions at UV and visible wavelengths. First, we obtain the BC volume fraction from AERONET retrieved *k* values at red and NIR wavelengths (670, 870, and 1020 nm) following the method of Schuster *et al*.^18^. Second, we apply Arola *et al*.^19^ method to calculate the BrC column volume fraction using UV-MFRSR retrievals of *k* at 368 nm (*k*_ret-368_) instead of 440 nm. The method assumes a mixture of BC and BrC components embedded in non-absorbing host and applies the Maxwell-Garnett (MG) mixing rule to calculate the effective imaginary refractive index (*k*_calc_), which depends on the volume fractions of BC (f_BC_) and BrC (f_BrC_) and their known *a priori* complex refractive indices (*k*_BC_ and *k*_BrC_). We calculate f_BrC_ by fitting *k*_calc_(f_BrC_, f_BC_, *k*_BrC_, *k*_BC_) to *k*_ret-368_ assuming lower and upper limits of *k*_BrC-368_ (see Fig. S1 in Supplement) and known fixed BC volume fraction (f_BC_ = 0.019) from step 1. Compared to shorter UV-MFRSR UV channels, the 368 nm channel has the advantage of large signal-to-noise (S/N), negligible gaseous absorption, and moderate Rayleigh optical thickness ~0.5 thus reducing uncertainties in UV-MFRSR *k* inversions.

The retrieval requires *a priori* knowledge of the BrC complex refractive index. The real part of refractive index, *n*_BrC_, is fixed similar to Arola *et al*.^19^. We determine the possible range of the imaginary part, *k*_BrC,_ from prior field and laboratory measurements for absorbing organic aerosols from biomass burning sources (*e.g.,* see Fig. S1 in Supplement). We interpolate prior *k*_BrC_ measurements to the 368 nm wavelength (*k*_BrC-368_) using least squares fitting to the power law spectral dependence (1):


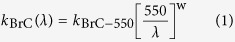


where *k*_BrC_ is the BrC imaginary refractive index and *k*_BrC-550_ is the measured BrC imaginary refractive index at reference wavelength (550 nm)^2^,^6^.

This range of *k*_BrC-368_ from 0.025 to 0.14 (Supplementary Fig. S1) together with the specific MG mixing rule assumption determines the possible range of calculated f_BrC_ and BrC/BC fraction ratio that are consistent with our retrievals of *k*: the larger the assumed value of *k*_BrC-368_, the smaller the inferred value of f_BrC_. We obtain the upper limit on f_BrC_ = 0.27 using the smallest reported *a priori* value of *k*_BrC-368_ ~0.025 from Saleh *et al*.^6^ and the lower limit value of f_BrC_ = 0.05 using the upper limit of *k*_BrC-368_ ~0.14 from Kirchstetter *et al*.^2^.

### BrC column mass density calculation

In order to derive column mass density for BC and BrC, we multiply their calculated volume fractions, f_BC_ and f_BrC_ by the AERONET-inferred total volume column density integrated over particle size distribution, and the assumed mass density (see equation (6) in Schuster *et al*.^18^ for BC and equation (2) in Arola *et al*.^19^ for BrC). We assume that the BrC mass density^40^ is 1.2 gm^−3^. Thus, lower and upper limits of the BrC column mass density are 10.9 and 61.2 mg/m^2^, while the average BC column mass density is 6.5 mg/m^2^ assuming a BC mass density^53^ equal to 1.8 gm^−3^.

### BrC spectral absorption calculation in UV

We derive *k*_BrC_ at UV wavelengths of 332, 325, 317, 311, and 305 nm by fitting UV-MFRSR retrieved *k*_ret_ assuming a fixed value of f_BrC_ = 0.05 (0.27) inferred from *k*_ret-368_ and known f_BC_ = 0.019 calculated from step 1 (Fig. 3). In other words, we modified the algorithm to infer *k*_BrC_ by fitting retrieved *k*_ret_ and *k*_calc_ at short UV wavelengths using fixed values of f_BC_ and f_BrC_ calculated in previous steps 1 and 2. Finally, we fit derived *k*_BrC_ at several UV-B and UV-A wavelengths to the power law equation (2) similarly to equation (1).


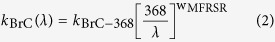


## Additional Information

**How to cite this article**: Mok, J. *et al*. Impacts of brown carbon from biomass burning on surface UV and ozone photochemistry in the Amazon Basin. *Sci. Rep.* **6**, 36940; doi: 10.1038/srep36940 (2016).

**Publisher’s note:** Springer Nature remains neutral with regard to jurisdictional claims in published maps and institutional affiliations.

## Supplementary Material

Supplementary Information

## Figures and Tables

**Figure 1:**
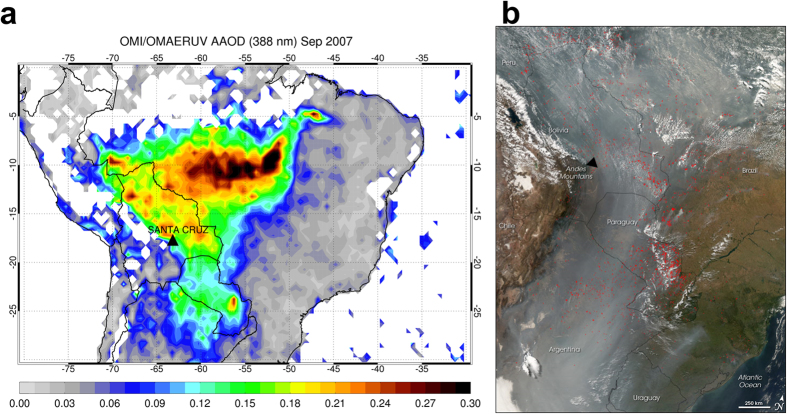
Distribution of biomass burning and resultant smoke over South America. (**a**) Satellite map of monthly mean aerosol absorption optical depth (AAOD = AOD*(1-SSA)) at 388 nm for September 2007 derived using two-channel OMAERUV aerosol algorithm applied to the Ozone Monitoring Instrument (OMI) on board NASA’s Aura satellite. The OMAERUV aerosol dataset is available from NASA’s Goddard Earth Sciences Data and Information Services Center (http://disc.sci.gsfc.nasa.gov/uui/datasets/OMAERUV_V003/summary?keywords=%2522Aura%20OMI%2522#prod-summary). This plot was created using the IDL (Interactive Data Language) software version 7.1.1. The URL link to access the IDL software is http://www.harrisgeospatial.com/ProductsandSolutions/GeospatialProducts/IDL/Language.aspx. (**b**) MODIS (Moderate Resolution Imaging Spectroradiometer) true-color image captured on 9 September 2007 over the same region showing active fire locations (marked in red) and a thick blanket of smoke stretching from the Amazon to Argentina; the image was obtained from NASA’s Earth Observatory website^54^. A solid black triangle shows the location of Santa Cruz.

**Table 1:** Estimated BrC mass absorption efficiency (MAE_BrC_).

Wavelength [nm]	Total AAOD	AAOD_BC_	AAOD_BrC_ = AAOD-AAOD_BC_	MAE_BrC_ [m^2^/g] (min [*k*_BrC-368_]^6^, max [*k*_BrC-368_]^2^)
305	0.296	0.121	0.175	(2.9–16.1)
311	0.233	0.120	0.113	(1.8–10.4)
317	0.255	0.117	0.138	(2.3–12.7)
325	0.189	0.115	0.074	(1.2–6.8)
332	0.212	0.114	0.098	(1.6–9.0)
368	0.156	0.102	0.054	(0.9–5.0)

We estimate MAE_BrC_ in the UV wavelengths (MAE_BrC_ = 0 in the visible wavelengths) using specific AAOD_BrC_ divided by BrC column mass density. The maximum and minimum values of MAE_BrC_ are associated with the range of previously measured *k*_BrC_^2^,^6^ at 368 nm (see Methods section for details).
