# Supplementary material for: Impacts of brown carbon from biomass burning on surface UV and ozone photochemistry in the Amazon Basin
Source: Sci Rep. 2016 Nov 11;6:36940. doi: 10.1038/srep36940 (PMC5105132; doi:10.1038/srep36940)
Supplement: Supplementary Information [file srep36940-s1.pdf]

Impacts of brown carbon from biomass burning on surface UV and ozone photochemistry in the Amazon Basin

Jungbin Mok[1,2], Nickolay A. Krotkov[3], Antti Arola[4], Omar Torres[3], Hiren Jethva[3], Marcos Andrade[5], Gordon Labow[3,6], Thomas F. Eck[3,7], Zhanqing Li[1,2*], Russell R. Dickerson[1,2], Georgiy L. Stenchikov[8], Sergey Osipov[8], and Xinrong Ren[1,9]

[1]Department of Atmospheric and Oceanic Science (AOSC), University of Maryland, College Park, Maryland, USA

[2]Earth System Science Interdisciplinary Center (ESSIC), College Park, Maryland, USA

[3]NASA Goddard Space Flight Center, Greenbelt, Maryland, USA

[4]Finnish Meteorological Institute, Kuopio, Finland

[5]Laboratory for Atmospheric Physics, Institute for Physics Research, Universidad Mayor de San Andres, La Paz, Bolivia

[6]Science Systems and Applications, Inc., Lanham, Maryland, USA

[7]Universities Space Research Association, Columbia, Maryland, USA

[8]Division of Physical Sciences and Engineering, King Abdullah University of Science and Technology, Thuwal, Saudi Arabia

[9]NOAA Air Resources Laboratory, College Park, Maryland, USA

1. AERONET and UV-MFRSR inversions

Our technique uses simultaneous measurements from co-located AERONET and UV-MFRSR instruments to ensure (1) accurate and consistent measurements of the spectral aerosol extinction optical depth (AOD), (2) consistent assumptions about surface reflectance, aerosol size distribution, and real part of the effective complex refractive index[1,2].

The primary data set consists of 1–minute measurements of diffuse and total irradiance at 305, 311, 317, 325, 332, 368, and 440 nm collected with the modified commercial UV-MFRSR instrument[3,4]. A single measurement cycle consists of measuring total horizontal irradiance (no sun blocking) following by 3 irradiance measurements with different positions of the shadow band blocking the sun and sky radiance on each side of the sun (at 9°). All spectral channels were measured within one second by 7 separate solid-state detectors with interference filters sharing a common Teflon diffuser. The complete shadowing cycle takes about 10 seconds and is repeated every minute throughout the day without averaging of the data. The raw data (voltages) were corrected for non-ideal angular response and calibrated against co-located reference AERONET sunphotometers.

The AERONET automatic tracking sun and sky scanning radiometers made direct sun measurements with a 1.2° full field of view every 15 minutes at 340, 380, 440, 500, 675, 870, 940, and 1020 nm. Its accuracy of AOD is typically ~0.003 to 0.01 in the visible with larger errors in the UV[5]. The AERONET version 2 level 2.0 AOD at 340, 380, 440, and 500 nm were interpolated in time and wavelength[6] and compared with the UV-MFRSR measurements of cosine corrected direct-normal voltages to derive daily V_0 calibrations, consistent AOD, and diffuse and direct atmospheric transmittances[1]. To ensure consistency between UV-MFRSR and AERONET retrievals, we only

consider UV-MFRSR inversions when derived AOD agrees with the interpolated AERONET AOD within 0.01.

We infer the column effective imaginary part of the refractive index (*k*) independently at 440 nm and each UV wavelength by fitting UV-MFRSR measured diffuse/direct (DD) irradiance ratios[7] with the Mie-RT modeled irradiances (Arizona code[8]). Ancillary input parameters, such as surface albedo, the column particle size distribution (PSD), and the real part of the refractive index (*n*), are taken from near simultaneous AERONET inversions (within 10 minutes). Absorption by trace gases (O_3 and NO_2) was accounted for using Aura/OMI satellite data. The Mie-RT model iterates to find the *k* value, which minimizes the difference between calculated and measured DD ratio. Details of approach are described in Krotkov *et al.*[2]. Requiring AERONET spherical particle fraction to exceed 95% validates the sphericity assumption.

2. OMI retrievals of carbonaceous aerosols

The Ozone Monitoring Instrument (OMI) flown onboard the EOS-Aura satellite as part of the A-train satellite constellation measures sun backscattered radiance from the Earth's atmosphere and surface in the UV and visible region (270 – 500 nm) on a global scale and on daily basis [9,10]. With a cross-track swath of about 2600 km, OMI scans the entire Earth in 14 to 15 orbits per day with a nadir ground pixel spatial resolution of 13 × 24 km^2. The primary objective of OMI is to measure concentrations of trace gases such as ozone, sulfur dioxide, and nitrogen dioxide along with the optical properties of tropospheric aerosols. OMI observations in the near-UV region at 354 nm and 388 nm are used to derive UV Aerosol Index (UV-AI) which is a well-accepted indicator for detecting and tracing the absorbing aerosols such as carbonaceous aerosols and dust[11-13] and to

retrieve AOD and SSA at 388 nm (OMAERUV product[10,14]). The most recent operational algorithmic upgrades are documented in Jethva and Torres[15] and Torres *et al.*[14] that include three major modifications: (1) improved representation of organic aerosols in the biomass burning smoke aerosols, (2) use of aerosol height climatology derived from Cloud-Aerosol Lidar with Orthogonal Polarization (CALIOP) lidar-based measurements of the vertical profiles in conjunction with OMI UV-AI, and (3) robust identification of aerosol types by taking advantage of concurrent observations of carbon monoxide (CO) measured by the Atmospheric Infrared Sounder (AIRS). The upgraded OMAERUV aerosol product has been validated globally using ground-based measurements of AOD[16] and SSA[17] inversions at 440 nm by the AERONET global network of ground sun-sky photometers. The comparisons of OMI-retrieved SSA against equivalent inversions made by AERONET revealed a good level of agreement between the two fundamentally different techniques in which most matchup data points are found to fall within their expected retrieval uncertainties (± 0.03) for SSA at 440 nm[17]. In the present study, we use the latest research version 1.7.3.1 of the OMAERUV aerosol product which includes major upgrades already documented in Jethva and Torres[15] and Torres *et al.*[14] plus a few more improvements that are mostly related to the assignments of retrieval quality flags.

3. Photolysis rate

Actinic fluxes in the atmosphere are computed in the 290 – 425 nm spectral range using the Discrete Ordinates Radiative Transfer Model (DISORT)[18] for a Multi-Layered Plane-Parallel Medium with the 16-stream setup. Chemical composition of the atmosphere is prescribed according to the monthly climatology derived from the Global Modeling Initiative (GMI) model output. The GMI 3-D chemistry and transport model is integrated with meteorological fields from the Modern

Era Retrospective-analysis for Research and Applications (MERRA) and includes full chemistry for both the troposphere and the stratosphere[19,20]. Gas absorption and scattering optical depths are computed using components from the Line-By-Line Radiative Transfer Model[21,22]. The aerosol optical properties (extinction, single scattering albedo, and phase function) are calculated for a given size distribution assuming the sphericity of particles using analytic Mie solution[23]. Lower boundary conditions assume a Lambertian reflection with the ultraviolet surface albedo of 0.04. Photolysis rate calculations are based on the absorption cross section and quantum yield data[24].

4. Box model

The box model is based on the Regional Atmospheric Chemical Mechanism Version 2 (RACM2)[25] and is run on the FACSIMILE platform for Windows software (MCPA Software, Ltd). RACM2 is well known and has been used in research and regulatory applications. The RACM2 mechanism with the chemical kinetic data replaced with the most recently evaluated set of chemical kinetic data[24,26] was used in this study.

RACM2 is a revised version of Regional Atmospheric Chemical Mechanism Version 1 (RACM)[27]. It is intended to be valid for conditions ranging from pristine to polluted and from the Earth's surface through the upper troposphere. The main revisions have been performed for organic chemistry such as the oxidation mechanisms for isoprene, α-pinene, and d-limonene. Most of the organic species are aggregated into the model species based on their similarity in functional groups and reactivity toward OH. For instance, alkenes other than ethene are represented by three species: terminal alkenes, internal alkenes, and dienes. Some organic species such as formaldehyde and isoprene are treated explicitly.

The box model was initialized with a NO_x (= NO + NO_2) concentration of 100 ppbv and a VOC concentration of 200 ppbv. To represent the smoke plume in this work, the mean NO to NO_2 ratio, VOC speciation, mean concentrations of long-lived species like CO and CH_4, as well as mean temperature, pressure and humidity in the Canadian wildfire plumes observed during the Arctic Research of the Composition of the Troposphere from Aircraft and Satellites (ARCTAS) in 2008 (espo.nasa.gov/arctas). The photolysis reaction coefficients, or photolysis frequencies, used in the model were calculated by radiative transfer model using DISORT with 16 streams (See Supplementary Section 3 for details). The box model was run for 20 days and the output calculated OH, HO_2, and other reactive intermediates. The net instantaneous O_3 production rate, $P(O_3)$, can be written approximately as the following equation:

$$P(O_3)=k_{HO_2+NO}[HO_2][NO] + \sum k_{RO_{2i}+NO}[RO_{2i}][NO] - k_{HO_2+O_3}[HO_2][O_3] - k_{OH+O_3}[OH][O_3] - k_{O(^1D)+H_2O}[O(^1D)][H_2O] \qquad (3)$$

where k terms are the reaction rate coefficients and RO_{2i} is the individual organic peroxy radicals. The negative terms correspond to the reaction the reactions of OH and HO_2 with O_3 and the photolysis of O_3 followed by the reaction of $O(^1D)$ with H_2O.

5. References

1. Krotkov, N. A. *et al.* Aerosol ultraviolet absorption experiment (2002 to 2004), part 1: ultra-violet multifilter rotating shadowband radiometer calibration and intercomparison with CIMEL sunphotometers. *Opt. Eng.* **44,** 041004 (2005).
2. Krotkov, N. A. *et al.* Aerosol ultraviolet absorption experiment (2002 to 2004), part 2: ab-sorption optical thickness, refractive index, and single scattering albedo. *Opt. Eng.* **44,** 041005 (2005).
3. Harrison, L., Michalsky, J. & Berndt, J. Automated multifilter rotating shadow-band radiom-eter: an instrument for optical depth and radiation measurements. *Appl. Opt.* **33,** 5118–5125 (1994).
4. Krotkov, N. A. *et al.* Aerosol column absorption measurements using co-located UV-MFRSR and AERONET CIMEL instruments. Proc. SPIE **7462,** Ultraviolet and Visible Ground- and Space-based Measurements, Trace Gases, Aerosols and Effects VI, 746205 (2009).
5. Holben, B. N. et al. An emerging ground-based aerosol climatology: Aerosol optical depth from AERONET. *J. Geophys. Res.* **106,** 12067–12097 (2001).
6. Eck, T. F. *et al.* Wavelength dependence of the optical depth of biomass burning, urban, and desert dust aerosols. *J. Geophys. Res.* **104,** 31333–31349 (1999).
7. King, M. D. & Herman, B. M. Determination of the ground albedo and the index of absorp-tion of atmospheric particulates by remote sensing. Part 1: theory. *J. Atmos. Sci.* **36,** 163–173 (1979).

8. Herman, B. M., Browning, R. S. & De Luisi, J. J. Determination of effective imaginary term of the complex refractive index of atmospheric dust by remote sensing: the diffuse-direct Radiation method. *J. Atmos. Sci.* **32,** 918–925 (1975).

9. Levelt, P. F. *et al.* The Ozone Monitoring Instrument. *IEEE Trans. Geosci. Remote Sens.* **44,** 1093–1101 (2006).

10. Torres, O. *et al.* Aerosols and surface UV products from Ozone Monitoring Instrument observations: An overview. *J. Geophys. Res.* **112,** D24S47 (2007).

11. Torres, O., Bhartia, P. K., Herman, J. R., Ahmad, Z. & Gleason, J. Derivation of aerosol properties from satellite measurements of backscattered ultraviolet radiation: Theoretical basis. *J. Geophys. Res.* **103,** 17099–17110 (1998).

12. Torres, O. *et al.* A Long-Term Record of Aerosol Optical Depth from TOMS Observations and Comparison to AERONET Measurements. *J. Atmos. Sci.*, **59,** 398–413 (2002)

13. Torres, O., Bhartia, P. K., Sinyuk, A., Welton, E. J. & Holben, B. Total Ozone Mapping Spectrometer measurements of aerosol absorption from space: Comparison to SAFARI 2000 ground-based observations. *J. Geophys. Res.* **110,** D10S18 (2005)

14. Torres, O., Ahn, C. & Chen, Z. Improvements to the OMI near-UV aerosol algorithm using A-train CALIOP and AIRS observations. *Atmos. Meas. Tech.* **6,** 3257–3270 (2013).

15. Jethva, H. & Torres, O. Satellite-based evidence of wavelength-dependent aerosol absorption in biomass burning smoke inferred from Ozone Monitoring Instrument. *Atmos. Chem. Phys.* **11,** 10541–10551 (2011).

16. Ahn, C., Torres, O. & Jethva, H. Assessment of OMI near-UV aerosol optical depth over land. *J. Geophys. Res.* **119,** 2457–2473 (2014).

17. Jethva, H., Torres, O. & Ahn, C. Global assessment of OMI aerosol single-scattering albedo using ground-based AERONET inversion. *J. Geophys. Res.* **119,** 9020–9040 (2014).

18. Stamnes, K., Tsay, S., Wiscombe, W. & Jayaweera, K. Numerically stable algorithm for discrete-ordinate-method radiative transfer in multiple scattering and emitting layered media. *Appl. Opt.* **27,** 2502–2509 (1988).

19. Strahan, S. E. *et al.* Using transport diagnostics to understand chemistry climate model ozone simulations. *J. Geophys. Res.* **116,** D17302 (2011).

20. Douglass, A. R. *et al.* Choosing meteorological input for the global modeling initiative assessment of high-speed aircraft. *J. Geophys. Res.* **104,** 27545–27564 (1999).

21. Clough, S. A. *et al.* Atmospheric radiative transfer modeling: a summary of the AER codes. *J. Quant. Spectrosc. Radiat. Transfer* **91,** 233–244 (2005).

22. Clough, S. A., Iacono, M. J., & Moncet, J. L. Line-by-line calculations of atmospheric fluxes and cooling rates: Application to water vapor. *J. Geophys. Res.* **97,** 15761–15785 (1992)

23. Veihelmann, B., Konert, M., & van der Zande, W. J. Size distribution of mineral aerosol: using light-scattering models in laser particle sizing. *Appl. Opt.* **45,** 6022–6029 (2006).

24. Sander, S.P. *et al.* Chemical Kinetics and Photochemical Data for Use in Atmospheric Studies. Evaluation No. 17. Jet Propulsion Laboratory, Pasadena, CA (2011).

25. Goliff, W. S., Stockwell, W. R., & Lawson, C. V. The regional atmospheric chemistry mechanism, version 2. *Atmos. Environ.*, **68,** 174–185 (2013).

26. Atkinson, R. *et al.* Evaluated kinetic and photochemical data for atmospheric chemistry: Volume IV – gas phase reactions of organic halogen species. *Atmos. Chem. Phys.* **8,** 4141–4496 (2008).

27. Stockwell, W. R., Kirchner, F., Kuhn, M., & Seefeld, S. A new mechanism for regional atmospheric chemistry modeling. *J. Geophys. Res.*, **102,** 25847–25879 (1997).

28. Saleh, R. *et al.* Brownness of organics in aerosols from biomass burning linked to their black carbon content. *Nature Geosci.* **7,** 647–650 (2014).

29. Saleh, R. *et al.* Absorptivity of brown carbon in fresh and photo-chemically aged biomass-burning emissions. *Atmos. Chem. Phys.* **13,** 7683–7693 (2013).

30. Chen, Y. & Bond, T. C. Light absorption by organic carbon from wood combustion. *Atmos. Chem. Phys.* **10,** 1773–1787 (2010).

31. Kirchstetter, T. W., Novakov, T. & Hobbs, P. V. Evidence that the spectral dependence of light absorption by aerosols is affected by organic carbon. *J. Geophys. Res.* **109,** D21208 (2004).

32. Chakrabarty, R. K. *et al.* Brown carbon in tar balls from smoldering biomass combustion. *Atmos. Chem. Phys.* **10,** 6363–6370 (2010).

33. Zhong, M. & Jang, M. Dynamic light absorption of biomass-burning organic carbon photo-chemically aged under natural sunlight. *Atmos. Chem. Phys.* **14,** 1517–1525 (2014).

34. Favez, O., Alfaro, S. C., Sciare, J., Cachier, H. & Abdelwahab, M. M. Ambient measurements of light-absorption by agricultural waste burning organic aerosols. *J. Aerosol Sci.* **40,** 613–620 (2009).

Figures

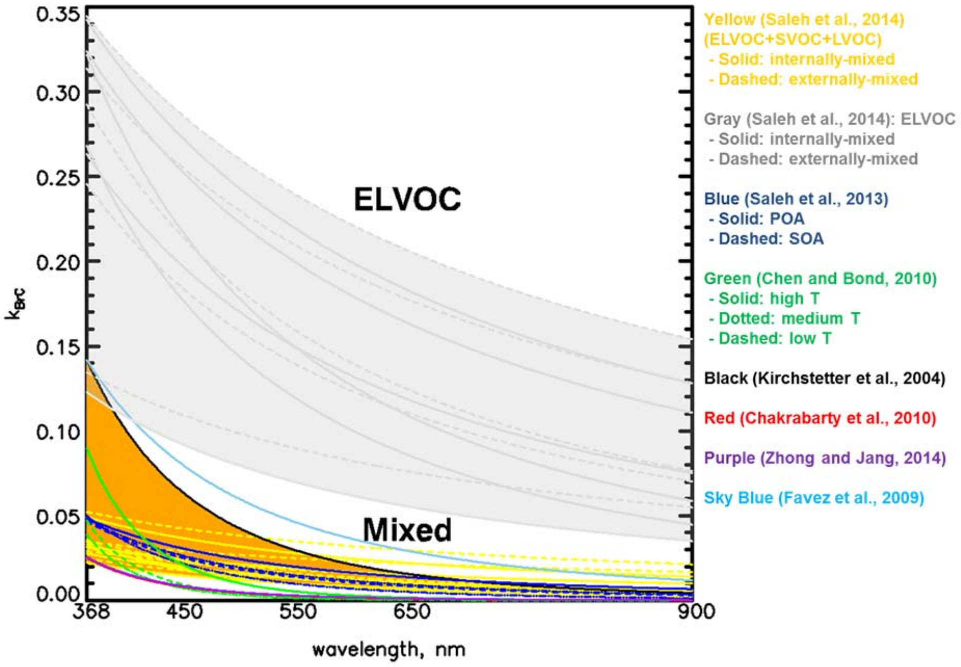

Figure 1. Summary of studies of brown carbon imaginary refractive indices for biomass burning. k_{BrC} from previous literature studies are parameterized by equation (1). POA and SOA correspond to primary organic aerosol and secondary organic aerosol, respectively. The topmost value of k_{BrC} at 368 nm ($k_{BrC-368}$ ~ 0.34) are found from smoke chamber experiments[28] shown as gray shade. However, we do not use this value because it is obtained by heating the aerosol to 250 °C to measure only highly absorptive extremely low-volatility organic compounds (ELVOC). Under typical conditions, ELVOCs are usually mixed with other OA components (semi-volatile organic compounds (SVOC) and low-volatile organic compounds (LVOC)). Excluding ELVOC, we use the highest $k_{BrC-368}$ (~0.14) in the mixed condition that Kirchstetter *et al.*[31] measured acetone soluble BrC extracted from African savanna biomass burning smoke samples. The lowest value of $k_{BrC-368}$ in the mixed condition is 0.025 measured by Saleh *et al.*[28]. The used range of $k_{BrC-368}$ is shown as orange shade.

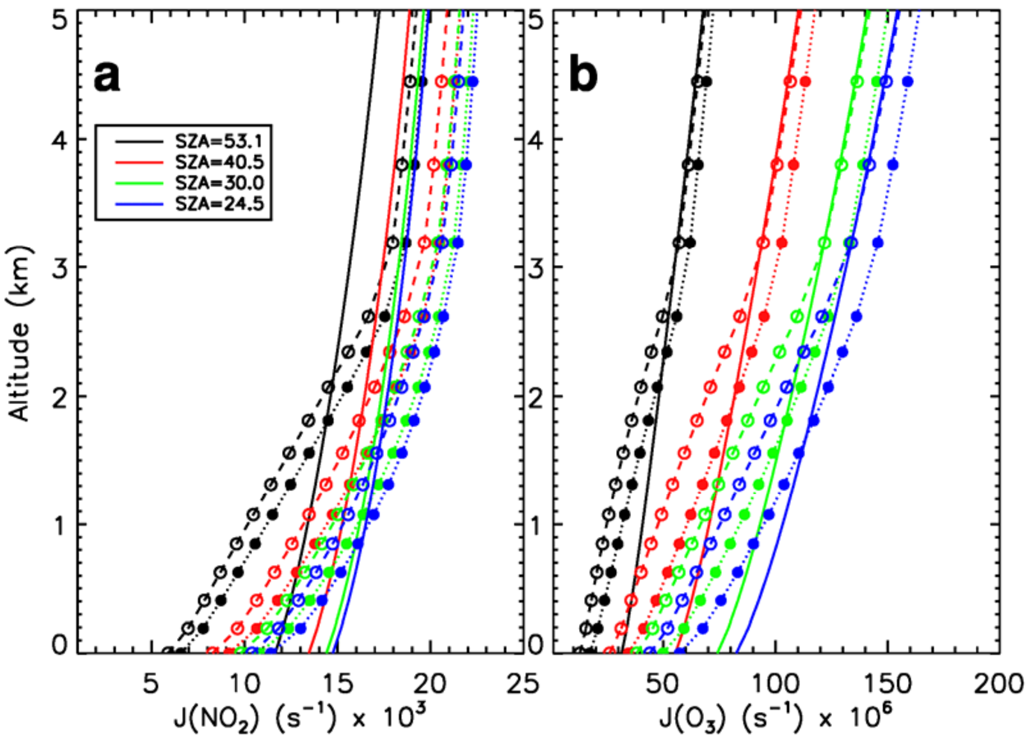

Figure 2. Model simulation for BrC effect on the photolysis rate. The vertical profile of **a,** J_{NO2} and **b,** J_{O3} from SZA=24.5° to SZA=53.1°. Solid line: no aerosol, dotted line with filled circle: BC only, and dashed line with open circle: BC+BrC (See Supplementary Table 2 for details). Comparing with the no aerosol case, J_{O3} and J_{NO2} for both BC only and BC+BrC cases show reduced values below the smoke layer due to the absorption of solar radiation but increased values above the smoke layer due to the higher backscattering. Interestingly, BrC causes not only more absorption below the smoke layer but also less backscattering above the layer than BC. In other words, BrC absorbs more UV flux in the boundary layer and cause less backscattering flux above the layer.

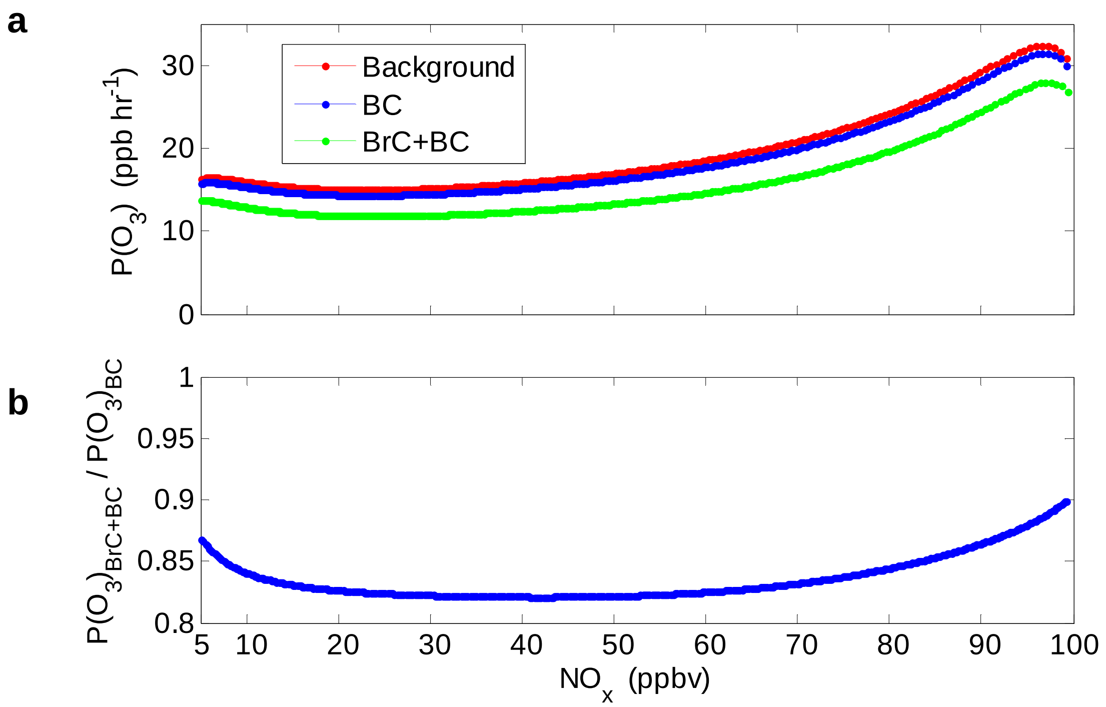

Figure 3. Modeling the impact of BrC absorption on surface ozone. a, Net ozone production ($P(O_3)$) in three cases: background (no aerosols), BC, and BrC+BC. **b,** The ratio of $P(O_3)$ in the BrC+BC case to $P(O_3)$ in the BC case. The box model was initialized with 200 ppbv VOCs and 100 ppbv NO_x. Both VOCs and NO_x are unconstrained in the model. The plotted NO_x range of 5 – 100 ppbv reflects ~2 – 3 days that are needed for a smoke plume to travel from the sources in Amazonia to Santa Cruz.

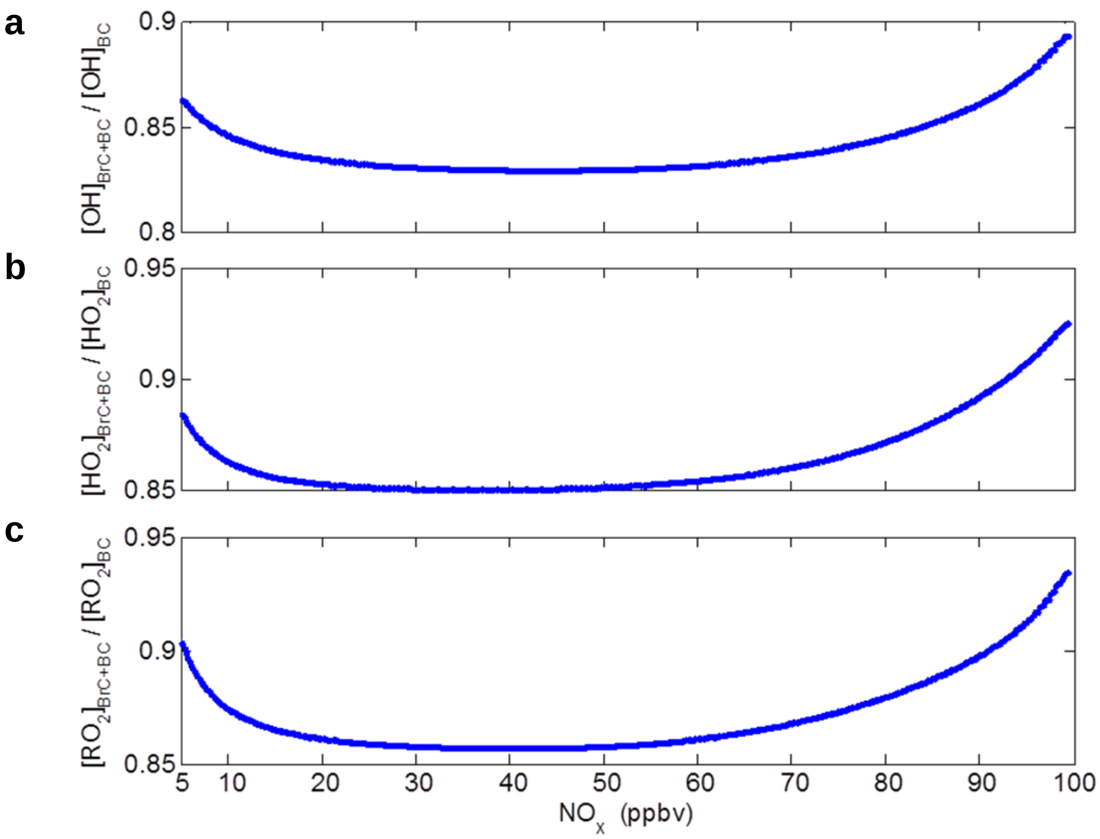

Figure 4. Modeling the impact of BrC absorption on OH, HO_2, and RO_2 concentration. a, The ratio of [OH] in the BrC+BC case to [OH] in the BC case, **b,** The ratio of [HO_2] in the BrC+BC case to [HO_2] in the BC case, and **c,** The ratio of [RO_2] in the BrC+BC case to [RO_2] in the BC case. The box model was initialized with 200 ppbv VOCs and 100 ppbv NO_x. Both VOCs and NO_x are unconstrained in the model. The plotted NO_x range of 5 – 100 ppbv reflects ~2 – 3 days that are needed for a smoke plume to travel from the sources in Amazonia to Santa Cruz.

Tables

Table 1. The calculated BrC volume fraction (f_{BrC}) and column mass density. The estimated ranges are the uncertainty bound.

Used k_{BrC} at 368 nm ($k_{BrC\text{-}368}$)	Kirchstetter *et al.*[31]	Saleh *et al.*[28]
BrC volume fraction	0.048	0.270
BrC column mass density ([BrC], mg/m^2)	10.89	61.24
[BrC]/[BC]	1.68	9.47

f_{BC} = 0.019 calculated from AERONET wavelengths (670, 870, and 1020 nm)

BC column mass density ([BC]) = 6.46 [mg/m^2]

Table 2. Summary of explicit input aerosol parameters for radiative transfer model and chemical model simulation.

Wavelength (nm)	305	311	317	325	332	368	440	670	870	1020
Model 1: *k* (BC only)	0.0126	0.0126	0.0126	0.0126	0.0126	0.0126	0.0126	0.0126	0.0126	0.0126
Model 2: *k* (BrC+BC)	0.0342	0.0271	0.0296	0.0209	0.0245	0.0192	0.0144	0.0125	0.0136	0.0122

median of real part of the refractive index at 440 nm from AERONET: 1.4905

median of surface albedo at 440 nm from AERONET: 0.04085

median of volume concentration for fine mode from AERONET: 0.1525

median of volume concentration for coarse mode from AERONET: 0.0365

median of (standard deviation from) volume median radius for fine mode from AERONET: 0.1810 (0.4320)

median of (standard deviation from) volume median radius for coarse mode from AERONET: 3.7410 (0.6585)
